# Evolution and fabrication of carbon dot-based room temperature phosphorescence materials

**DOI:** 10.1039/d3sc00062a

**Published:** 2023-03-02

**Authors:** Jiurong Li, Yongzhong Wu, Xiao Gong

**Affiliations:** a State Key Laboratory of Silicate Materials for Architectures, Wuhan University of Technology Wuhan 430070 P. R. China xgong@whut.edu.cn; b School of Mechanical Engineering, Suzhou University of Science and Technology Suzhou 215009 P. R. China

## Abstract

Traditional room temperature phosphorescence (RTP) materials usually include organometallic composites and pure organic compounds, which generally possess the disadvantages of high toxicity, high cost and complicated preparation. Carbon dots (CDs) are a new kind of luminescent material and have attracted widespread attention due to their benefits of excellent tunable emission, nice biocompatibility, cost-effectiveness, facile preparation and environmental friendliness. Since photoluminescence is an important luminescent property of carbon-based fluorescent nanomaterials, CD-based RTP materials have sparked a new research wave due to the properties of extremely long phosphorescence lifetime, large Stokes shift and high environmental sensitivity. In order to construct excellent CD-based RTP materials, many attempts have been made, and the corresponding progress has been achieved. Herein, we summarize the progress in CD-based RTP materials in recent years, mainly focusing on the outstanding contributions over the years, phosphorescence emission, phosphorescence lifetime, preparation and application of CD-based RTP materials. In particular, this review provides a comprehensive summary and analyze the outstanding contributions in the fields of the phosphorescence emission and phosphorescence lifetime of CD-based RTP materials over the years. Finally, several existing challenges and the future outlook of RTP materials based on CDs have been put forward.

## Abstract 2

Carbon dot-based room temperature phosphorescence materials are reported, mainly focusing on the outstanding contributions over the years, phosphorescence emission, phosphorescence lifetime, preparation and application.

## Graphical abstract

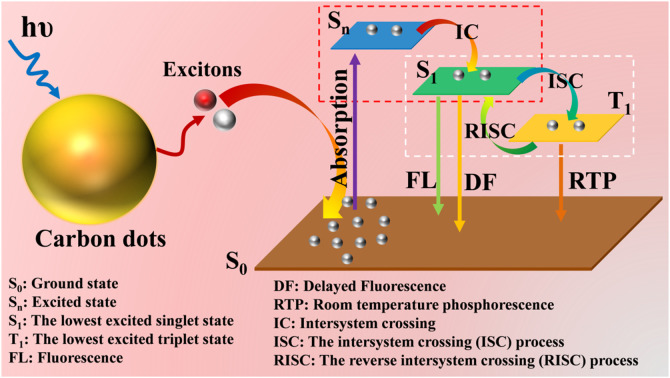

## 1. Introduction

Since the discovery of carbon dots (CDs) in 2004^1^ and after nearly twenty years of development, researchers have put many efforts towards understanding them. As a new class of zero-dimensional fluorescent nanomaterials, CDs have attracted widespread concern and great research interest from a wide range of researchers owing to their distinguished properties such as adjustable photoluminescence (PL), excellent stability, facile surface functionalization, environmental friendliness, low toxicity and good biocompatibility.^2–4^ According to previous reports,^5^ CDs can be classified into four types, namely carbon nanodots (CNDs), carbon quantum dots (CQDs), graphene quantum dots (GQDs) and carbonized polymer dots (CPDs). Specifically, GQDs are small graphene fragments less than 20 nm in lateral dimension and less than five graphene flakes (∼2.5 nm) in height, exhibiting pre-existing graphitic domains (sp^2^ domains) and edge-rich chemical groups. CQDs are morphologically quasi-spherical carbon nanoparticles with a distinct lattice and chemical groups on their surface, and they have a crystalline core based on a mixture of sp^2^ and sp^3^ domains. CNDs are also defined as quasi-spherical carbon nanoparticles, which consist mainly of cores with an amorphous structure, *i.e.*, CNDs have a certain degree of carbonization. CPDs have a hybrid polymer/carbon structure formed through the aggregation or cross-linking of linear polymers or monomers, with a surface consisting of abundant functional groups/polymer chains and carbon cores. Up to now, some notable achievements have been obtained in CD-related research, including the enhancement of photoluminescence quantum yield (PLQY), the regulation of multicolor fluorescence, the study of the luminescence mechanism and potential multifunctional applications.^6–14^ More importantly, the selection of different carbon source precursors and synthesis methods will lead to different fluorescence properties. The fluorescence of CDs is only one of the most common phenomena in their luminescent properties. In addition, CDs also possess other types of excellent luminescent properties, such as room temperature phosphorescence (RTP), thermally activated delayed fluorescence (TADF), up-conversion luminescence (UCL), chemiluminescence (CL), electrochemical luminescence (ECL), mechanoluminescence and so on.^15^ These luminescent properties have shown significant application prospects in biomedicine, optoelectronic devices, energy storage, sensing, anti-counterfeiting, catalysis, imaging and other fields.^16–19^ Particularly, the RTP phenomenon of CDs has afterglow emission performance which is very attractive because of its prolonged emission lifetime, larger Stokes shift and minimized interference from short-lived auto-fluorescence and scattered light. Now it has shown a broad application prospect in anti-counterfeiting, information encryption, sensing and other advanced fields.

**Fig. 1:**
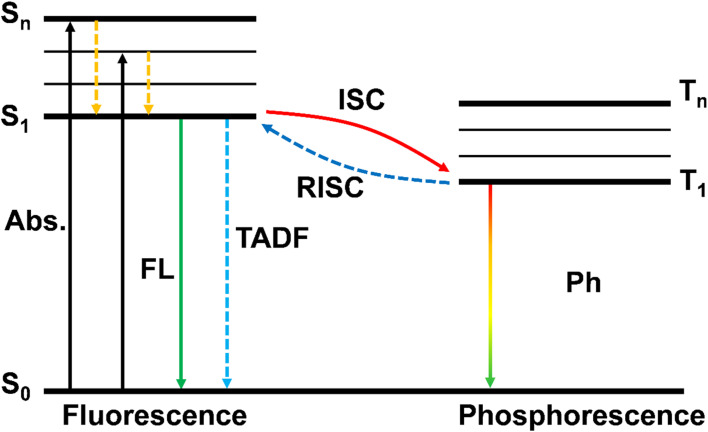
Schematic illustration of the adsorption and emission processes of fluorescence (FL), phosphorescence (Ph), and delayed fluorescence (DF).

In the last twenty years, the research on CDs has been widely carried out, and people have also reviewed and summarized their fluorescence in various aspects, such as the classification, synthesis methods, fluorescence regulation, fluorescence mechanism, different precursor selection, and applications in various fields.^6,8,10,14,20–34^ At the same time, the corresponding prospects for the fluorescence development of CDs are also proposed. Although there are some reviews providing a brief introduction of the afterglow phenomenon (*i.e.*, RTP and TADF) of CDs,^15–19,35–38^ there are few articles specifically describing the afterglow phenomenon related performance in detail.^39,40^ Based on the results previously reported, the radiative transition of triplet excitons is the key factor of the long afterglow phenomenon produced by CDs. The phosphorescence of CDs needs to be generated and enhanced by heteroatom doping or by embedding CDs into host matrices (*e.g.*, boric acid, polyvinyl alcohol, inorganic salts, layered double hydroxides, and zeolites) or by immobilizing CDs on a substrate. These matrices are essential for stabilizing the long-lived ternary state of CD fluorescence by effectively isolating and rigidifying CDs, minimizing the non-radiative leap of the triplet exciton and avoiding collisions of CDs with oxygen. In order to better distinguish the principal difference between fluorescence and phosphorescence, as shown in Fig. 1, the ground state (S_0_) transits to the lowest singlet excited state (S_1_) after being absorbed by ultraviolet and visible light, and then re-radiates back to S_0_ and emits fluorescence. The generated excitons first enter S_1_ from S_0_, and then enter the lowest triplet state (T_1_) through the intersystem crossing (ISC) process, and the excitons return to S_0_ again and generate phosphorescence through the process of radiation transition. Or the exciton first goes through the process of reverse intersystem crossing (RISC), returns to S_1_ from T_1_, and then returns to S_0_ to generate delayed fluorescence. Obviously, since the energy of T_1_ is lower than that of the singlet state S_1_, the phosphorescent emission peak will be significantly red shifted compared with fluorescence. It is worth noting that the fluorescence phenomenon of CDs is easy to realize, but the RTP of CDs is much harder to achieve. This is mainly the result of the intrinsic properties of the triplet excitons in relation to the spin-forbidden transition. More importantly, the triplet exciton is readily exhausted by vibrational deactivation and oxygen quenching under room temperature or higher. Thus, it can be seen that in order to obtain effective RTP, two important conditions need to be met, namely to improve the rate of ISC by enhancing the spin–orbit coupling of excitons and to stabilize the triplet-excited states *via* using structural confinement. For the first condition, it is generally possible to enhance the n–p* transition by incorporating transition metals, halogens or heterocycle groups to promote the ISC process and further generate triplet excitons. The second condition can be well achieved by encapsulating CDs in various substrates or creating self-protective structures (without substrate immobilization). Up to now, some efforts have been made to realize CD-based RTP materials to further explore their potential applications. Although some achievements have been made in the synthesis, construction and application of CD-based RTP materials, so far no article has systematically and completely summarized and analyzed them. And also, there have been several reviews on CD-based RTP materials in recent years, but they are only summarized and analyzed unilaterally for the synthesis, application or construction of CD-based RTP materials. Therefore, this review provides a summary and analyzes comprehensively CD-based RTP materials, and systematically classifies and analyzes the relevant published academic papers. From the first discovery of CD-based RTP materials to the current research, it summarizes and analyzes some important research in each year, especially the phosphorescence life study of CD-based RTP materials.

## 2. Synthesis of CD-based RTP materials

**Fig. 2:**
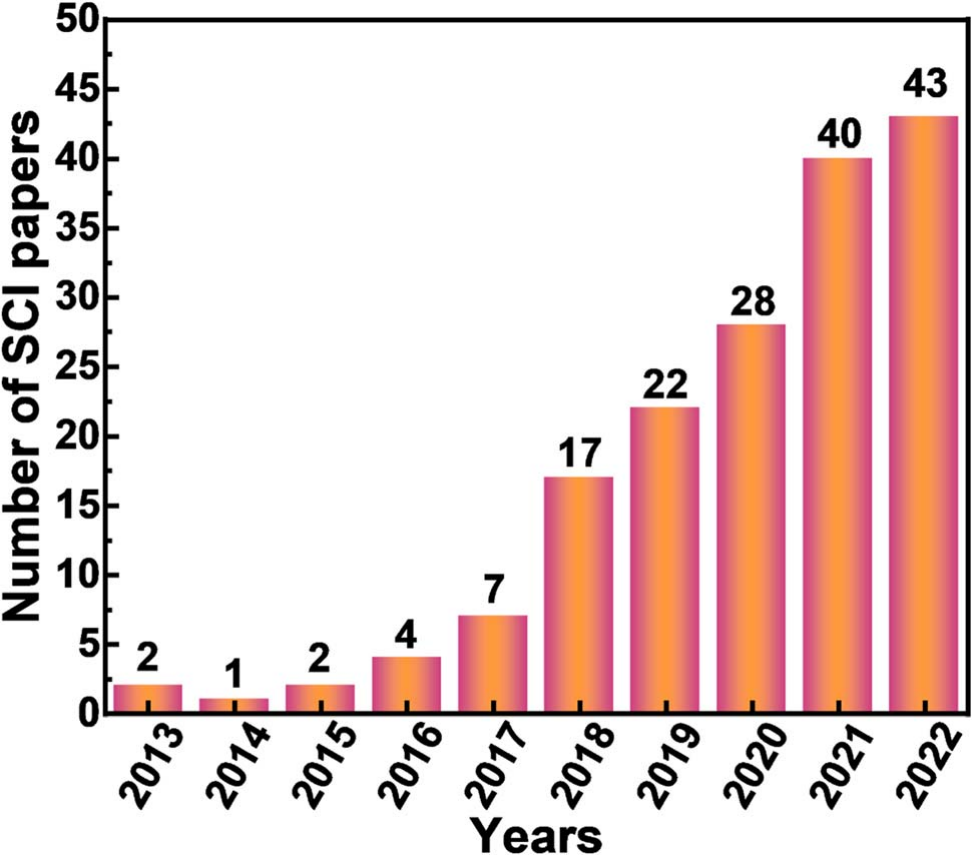
The number of articles related to CD-based RTP since 2013; search results were obtained from the Web of Science, on 31st August 2022.

Since the first report on CDs in 2004,^1^ a variety of preparation methods have been reported successively, which can be mainly divided into two categories through summary analysis, namely, top-down approach and bottom-up approach.^8,10,21,25–29,41^ As a special characteristic of CD-based luminescent materials, RTP has also been developed and reported. A variety of methods have also been reported for the synthesis of CD-based RTP materials, and they can also be mainly divided into two categories, namely matrix-assisted method (Table 1) and self-protection method (Table 2). The matrix-assisted method refers to the generation and enhancement of CD RTP by embedding them in a host matrix (*e.g.* boric acid, polyvinyl alcohol, layered double hydroxides, zeolites, *etc.*) or by immobilizing them on a substrate. This method has a wide range of practicality and can achieve the RTP properties of most CDs, but most of this method requires the use of multiple steps and harsh experimental conditions, often requiring high temperatures to achieve. The self-protection method means that the RTP properties of CDs are achieved by self-doping. This method is convenient, simple and highly scalable, but it is not suitable for large-scale preparation and it is difficult to find suitable precursors. At present, the most popular method is matrix-assisted synthesis. To date, in the preparation of metal free RTP materials, especially carbon-based RTP materials (mainly represented by CDs), more and more attention has been paid. The number of papers published is also increasing year by year, as shown in Fig. 2. As of August 31, 2022, a total of 166 articles related to CD-based RTP had been published through the keyword search of the paper. It is worth affirming that there may be omissions in the process of literature retrieval. We are sorry if some literature has not been retrieved.

**Table 1:** Matrix-assisted CD-based RTP materials^a^

Matrix	Precursor	Afterglow mode (298 K)	Afterglow emission (nm)	Afterglow lifetime (*τ*) ms^−1^ (298 K)	PQY (%)	Remarks	Ref.
PVA	CDs and PVA	RTP	∼500	380	NA	CDs–PVA	42
		RTP + TADF	569	1610	NA	CDs/PVA	43
		RTP	422	338.4	6.88	CDs-OH/PVA	44
		RTP + TADF	435	567	6.24	CNDs – PVA	45
			505	1387	12.32		
			560	726	4.32		
			590	311	2.03		
KAl(SO_4_)_2_·*x*(H_2_O)	CDs and KAl(SO_4_)_2_·*x*(H_2_O)	RTP	500	655	NA	CD-KAl(SO_4_)_2_·*x*(H_2_O)	46
Urea	CDs and urea	RTP	490	1.06	7%	HN powder	47
Urea	CDs and urea	RTP + DF	500	1020	7.23	NCD1-C	48
			502	1110	14.0	NCD2-C	
			625	780	11.0	NCD3-C	
PU	N-doped CQDs and PU	RTP + DF	500	8.7	NA	CQD/PU	49
LDHs	CDs and EDTA-LDHs	RTP	525	386.8	5.99	CDs-LDHs	50
	CDs and LDHs (Zn^2+^)	RTP	490	800	9.44	Zn-CDs-LDH	51
	CDs and LDHs (Zn^2+^)	RTP	490	719.9	9.58	Zn-CDs-LDH	52
SiO_2_	m-CDs and colloidal nanosilica	RTP + TADF	470	703	NA	m-CDs@nSiO_2_	53
	CDs and silica nanoparticles	RTP	520	1640	NA	CDs@SiO_2_	54
	CDs and nanosilica	RTP + TADF	508	1760	NA	CDs@SiO_2_	55
	CDs and silica	RTP + TADF	507	1620	NA	CDs@SiO_2_	56
	CDs and the SiO_2_ matrix	RTP	464	5720	26.36	CDs@SiO_2_	57
	CDs and silica	RTP	520	1860	11.6	WSP-CNDs@silica	58
	CNDs, Rhodamine B and silica	RTP	600	910	3.56	CNDs-RhB@silica	59
	CNDs and tetraethoxysilane	RTP	520	1570	12.6	CNDs@silica	60
	CDs, fluorescent dyes and amorphous silica	RTP	510 to 610	1610 to 1260	NA	PCDs	61
Silica gel	CDs and silica gel	RTP	525	1800	NA	CDs in silica gel	62
Zeolites	CDs and a series of zeolites	RTP + TADF	430	350	NA	CDs@zeolite	63
	CDs and zeolite	RTP	500	22.32	14.1	CDs@zeolite	64
			620	1.81	5.7		
	CDs and zeolite	RTP + TADF	525	574	NA	CDs@zeolite	65
			440	153			
	CDs and zeolite	RTP + TADF	530	2100	24.4	CDs@zeolite	66
	CDs, Eu^3+^ and AlPO4-5 zeolite	RTP + TADF	516	1400	NA	CDs@EuAPO-5	67
	CDs and zeolite	RTP	516–520	380–2100	NA	CDs@zeolite	68
NaCl	CDs and NaCl	RTP	519	314	NA	CD–NaCl	69
Cyanuric acid	CDs and cyanuric acid	RTP	480	705	14	CD–CA	70
		RTP	480	687	NA	CD–CA	71
		RTP + TADF	550	220.74	NA	CD@CA	72
			690	13.29			
	CDs and urea	RTP	425–510	>2 hour	NA	m-CDs@CA	73
	CDs and cyanuric acid	RTP	416 and 550	1740	23.2	m, p-CDs@CA	74
ZnAl_2_O_4_	CDs and zinc aluminate	RTP	517–650	NA	NA	CDs–ZnAl_2_O_4_	75
Boric acid	CDs and boric acid	RTP	490–570 nm	2.26	17.5	a-CDs/BA	48
	CDs and boric acid	RTP + TADF	454–500	783	NA	GQD@BNO	76
	Lycorine hydrochloride and boric acid	RTP	520	1760	30	CDs/BA	77
	CDs and boric acid	RTP + TADF	475–555	445.9	17.61	CDs/B_2_O_3_	78
	CDs and boric acid	RTP	530	1670	48	g-t-CD@BA	79
MOF	CDs and MOF	RTP	478–631	85.67–1064.21	6.5–18.6	CDs@MOF	80
Mn-framework	CDs and Mn-framework	RTP	530	10.48	NA	CDs@MnAPO-CJ50	81
			620	10.94	9.6	CDs@MnAPO-tren	
Melamine	CDs and melamine	RTP	529	664	25	M-CDs	82
Clay	CDs and clay	RTP + DF	450	1050	6.06	CDs@clay	83
			471	1020	8.27		
			530	608	1.08		
Polyvinylpyrrolidone	CDs and PVP	RTP	580	582	NA	CD/PVP	84
Cellulose	CDs and cellulose fibrils	RTP	560	167.31	NA	CDs@cellulose	85

a NA: not available; PQY: phosphorescence quantum yield; TADF: thermally activated delayed fluorescence; RTP: room temperature phosphorescence; DF: delayed fluorescence; *m*PDs: *m*-phenylene diamine; MOF: metal–organic framework; PVA: polyvinyl alcohol; PVP: polyvinylpyrrolidone; PU: polyurethane.

**Table 2:** Self-protection CD-based RTP materials^a^

Method	Precursor	Afterglow mode (298 K)	Afterglow emission (nm)	Afterglow lifetime (*τ*) ms^−1^ (298 K)	PQY (%)	Remarks	Ref.
Hydrothermal	Citric acid and ethylenediamine	RTP	440	0.16	NA	CDs	86
Hydrothermal	PVA and ethylene diamine	RTP	564	13.4	NA	NCD	87
Hydrothermal	Polyacid and diamine	RTP	494	658.11	NA	CDs	88
Microwave-assisted	Ethanolamine and phosphoric acid	RTP	535	1460	NA	URTP CDs	89
Heat treatment	Laspartic acid and d-glucose	RTP	515	747	NA	NCDs	90
Solvothermal	Glucose and (C_2_H_5_)_3_N·3HF	RTP	455	1210	3.45	FNCDs	91
Microwave-assisted	Triethanolamine and phosphoric acid	RTP	518	820	15.85	P-CDs	92
Hydrothermal	Acrylamide, urea and citric acid	RTP	519	459	NA	NCDs	93
Heat treatment	Urea and phosphoric acid	RTP	495	320	23	SW-CPDs	94
Hydrothermal	Isophthalic acid and ethylenediamine	RTP	510	769	NA	N-CDs	95
Carbonization	Citric acid and 2,4,6-trihydrazinyl-1,3,5-triazine	RTP	476	66.4	4.7	C-dots	96
Hydrothermal	Citric acid (CA) and basic fuchsine	RTP	550	51.9	28	N-CDs	97
Solvent-free pyrolysis treatment	Hydroxyurea	RTP	570	175	13	FP-CDs	98
Solvothermal	5,5′-Disulfanediylbis(2-nitrobenzoic acid)	RTP	426/520	1.1	NA	HCDs	99
Solvothermal	Phenol, cytosine, resorcinol, and phloroglucinol	RTP	500	65	NA	F, O-codoped CDs	100
			505	127			
			520	218			
Microwave-assisted	Polyethylenimine and phosphoric acid	RTP	515	565.19	NA	N-CDs	101
Solvothermal	Fructose and diethylenetriamine	RTP	500	1140	8.3	FNCDs	102
Hydrothermal	Diethylenetriamine, phosphoric acid and boric acid	RTP	509	481	8.7	CPDs	103
			535	511	6.3		
			567	437	3.2		
			603	426	1.5		
Hydrothermal	Levofloxacin	RTP	555	354	4.2	CD@paper	104
			630	237			
Solvothermal	Urea, HCl, citric acid, DMF	RTP + DF	500	314	5.8	CD powder	105
			572	462			
One-step melting	Boric acid	RTP	512	1740	66.13	(NACA)_0.01%_/BA	106
			495				
			560				
Hydrothermal	Silica and hexamethyleneimine	RTP + TADF	508	451	NA	CDs@SiO_2_	107
Solvent-free catalytic assistant	*o*-Phenylenediamine and AlCl_3_·6H_2_O	RTP	554	243.2	2.8	M-CDs	108
Pyrolysis	Citric acid and boric acid	RTP	466–638	113.9–581.76	0.42–13.74	B-CD	109
Microwave-assisted	1-[3-(Trimethoxy silyl) propyl]urea and phosphoric acid	RTP	480–560	1000	59.41	CPDs	110

a NA: not available; PQY: phosphorescence quantum yield; TADF: thermally activated delayed fluorescence; RTP: room temperature phosphorescence; DF: delayed fluorescence; PVA: polyvinyl alcohol.

### 2.1 Matrix-assisted synthesis of CD-based RTP materials

The matrix-assisted approach involves embedding CDs into appropriate matrix structures, such as polymeric matrices, crystalline structures, mesoporous structures, inorganic layer structures, *etc.* These matrices can provide a strong rigid environment, dense hydrogen bonding sites or strong covalent bonds that can lock their excited triplet states and suppress their non-radiative relaxation, resulting in RTP emission. To date, most CDs-based RTP has been achieved by embedding a number of matrices (Table 1), and many RTP CD-based matrix composites have been discovered by combining the outstanding PL properties of CDs with the effective confinement effects of various matrices.

**Fig. 3:**
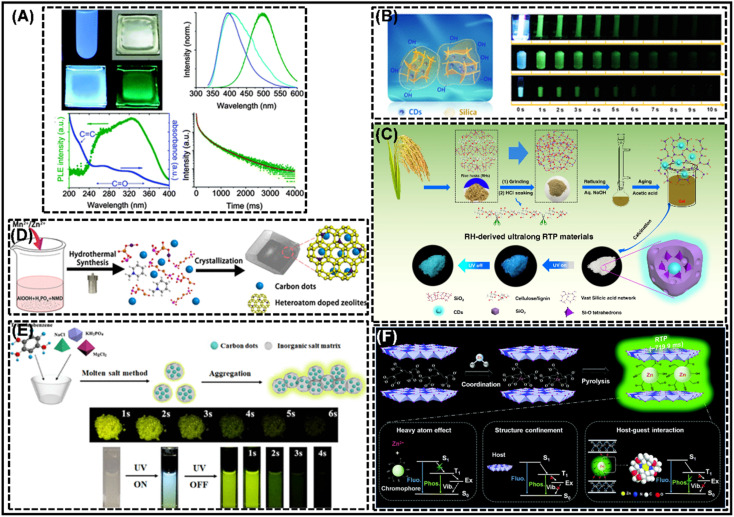
Matrix-assisted synthesis of CD-based RTP materials. (A) Reprinted with permission from Deng *et al.*^42^ Copyright 2013, Royal Society of Chemistry. (B) Reprinted with permission from Li *et al.*^54^ Copyright 2019, American Chemical Society. (C) Reprinted with permission from Sun *et al.*^57^ Copyright 2019, Springer. (D) Reprinted with permission from Wang *et al.*^64^ Copyright 2019, American Chemical Society. (E) Reprinted with permission from Wang *et al.*^124^ Copyright 2019, American Chemical Society. (F) Reprinted with permission from Kong *et al.*^52^ Copyright 2019, Royal Society of Chemistry.

Polymers are considered ideal matrices because of their rich functional groups and long regular chains, which not only provide stable chemical bonds with CDs, but also have the effect of separating solvent and oxygen and thus avoiding aggregation-induced fluorescence quenching. In particular, polyvinyl alcohol (PVA) was the first matrix material utilized to achieve CD-based RTP, which is currently the most commonly employed matrix support material.^42–45,111–117^ In 2013, Deng *et al.*^42^ first reported the observation of phosphorescence emission by compounding CDs with the PVA matrix (Fig. 3A). It is found that the PVA matrix plays a key role in protecting triplet states with long life from being effectively quenched by non-radiative processes, because it has a large number of hydrogen bonds that can effectively lock the emitting species and inhibit their intramolecular motions, *i.e.*, a non-radiative relaxation channel. In addition, PVA molecules also have a large number of hydroxyl groups, which can effectively form hydrogen bonds, making the C=O bond on the surface of CDs rigid, limiting the intramolecular motions and preventing non-radiative relaxation. Besides, oxygen is a strong quencher of the triplet state, but PVA has excellent oxygen barrier properties. Therefore, another potential function of PVA is to effectively prevent the direct collision between aromatic carbonyls and oxygen molecules, thereby promoting phosphorescence. Mesoporous materials are also a suitable class of matrix-assisted materials for achieving the RTP properties of CDs, which are a class of amorphous, ordered or crystalline materials with pore sizes of 2–50 nm that are good host matrices, and capable of accommodating luminescent species. As such, it is capable of accommodating CDs to achieve RTP, particularly silica, and is widely applied for the generation of CD RTP.^53–60,107,118^ For example, Li *et al.*^54^ proposed a strategy for achieving ultra-long RTP in air-saturated aqueous media based on carbon dot-based silica composites (CDs@SiO_2_) (Fig. 3B). The formation of Si–O–C bonds between CDs and silica during TEOS hydrolysis acts as a scaffold for the nucleation and the growth of the silica framework. The CDs are able to covalently attach to the silica network and the silica acts as a matrix that contributes to the dispersion of the CDs, providing protection from environmental bursts such as water and oxygen. More importantly, the abundance of silanol groups on the surface of the composites gives the whole hybridized system a good hydrophilic character. And also, Sun *et al.*^57^ designed and developed a strategy to fabricate metal-free multi-confined CDs (CDs@SiO_2_) within SiO_2_ by generating an effective multi-confinement effect (MCE) to develop room temperature phosphorescent materials with simultaneous ultra-long lifetime, high phosphorescence quantum efficiency and excellent stability (Fig. 3C). In addition, zeolites are a class of matrix-assisted materials widely utilized to achieve ultra-long phosphorescence lifetimes, and they are crystalline aluminosilicates or aluminophosphates with a three-dimensional 4-connected structure and a uniform pore size of less than 2 nm.^63–68,119^ For instance, Wang *et al.*^64^ have achieved a facile strategy to modulate the RTP properties of CDs through donor–acceptor energy transfer in the CDs–zeolite system by introducing heteroatoms into the aluminium phosphate zeolite backbone to construct an efficient donor–acceptor system that promotes exchange coupling between the CD exciton and the dopant in the matrix (Fig. 3D). Furthermore, the confinement effect of the crystal structure not only immobilizes T_1_ but also prevents non-radiative relaxation of T_1_ and is also a promising method for generating CD-based RTP. Crystal structures can be divided into two categories: organic crystal structures and inorganic crystal structures. The organic crystal structures include urea, cyanuric acid and melamine, while inorganic crystal structures include sodium chloride, sodium hydroxide, boric acid and so on.^46–48,70,72–76,78,79,82,120–130^ For example, Wang *et al.*^124^ proposed a molten salt method for the preparation of CD-based RTP materials by selecting high charge density magnesium salts and phosphates as doping salts, and calcining the carbon source directly in the presence of inorganic salts (Fig. 3E). During the melting and recrystallization process, CDs are formed and incorporated into a matrix with a special crystal structure. As magnesium phosphate is insoluble in water, the solid matrix provides rigid protection for the CDs. As a result, the CDs are non-phosphorescent as monomers, but the RTP phenomenon is initiated and enhanced by the aggregation of the salt matrix. Besides this, structural confinement displays a unique advantage by reducing the rate of the radiative relaxation process. A number of special structural matrices have also been used for the realization of CD RTP, such as two-dimensional (2D) layered double hydroxides (LDHs) which have attracted significant interest in the available confinement matrix owing to their versatile chemical composition and layer charge. Significantly, LDHs have a nanoscale space that provides a constrained and rigid environment for a number of chromophores that exhibit enhanced fluorescence properties (intensity, efficiency, lifetime, *etc.*) by suppressing the non-radiative relaxation of single-linear excitons.^52^ Therefore, exploring the relationship between the relaxation paths of triplet-state excitons and the rigidity of LDHs will help to achieve optimal luminescence efficiency and to develop a new class of RTP materials. Kong *et al.*^52^ employed LDHs as a matrix and proposed the design principle of activation to achieve RTP on CDs through three synergistic effects (structure-bound effect, heavy atom effect, and chemical bonding) (Fig. 3F). The confined and rigid environment of LDHs suppressed the non-radiative deactivation of CD triplet excitons and improved the luminescence efficiency. In conclusion, in addition to the above matrix-assisted materials, there are also other appropriate matrix-assisted materials suitable for achieving RTP of CDs. Therefore, the appropriate selection of matrices is key to achieving efficient and ultra-long RTP CDs.

### 2.2 Self-protection synthesis of CD-based RTP materials

Although the RTP of CDs in solid and water-disperse states has been achieved, the CD-based RTP materials prepared by matrix-assisted methods are mostly heterogeneous systems with poor thermal stability and electrical conductivity, which hinders their practical application. Therefore, to overcome these issues, the development of matrix-free self-protected phosphorescent CDs is an urgent problem to be solved. Aiming to generate self-protected RTP of CDs, the choice of the raw material is crucial. Currently, researchers often choose polymers or their monomers, cross-linkable reactants, molecules doped with heteroatoms and larger conjugated structures as precursors.^86–96,98–109,131,132^ The relevant achievements are shown in Table 2.

**Fig. 4:**
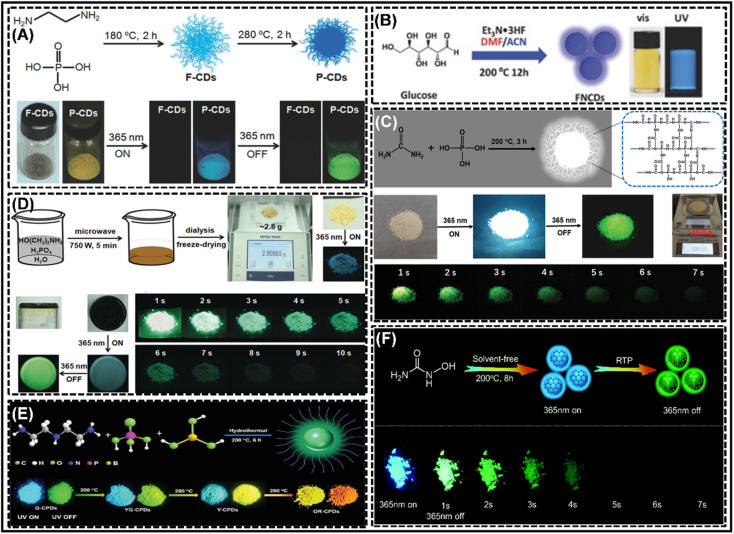
Self-protection synthesis of CD-based RTP materials. (A) Reprinted with permission from Jiang *et al.*^131^ Copyright 2018, Wiley-VCH. (B) Reprinted with permission from Long *et al.*^91^ Copyright 2018, Wiley-VCH. (C) Reprinted with permission from Wang *et al.*^94^ Copyright 2020, Wiley-VCH. (D) Reprinted with permission from Jiang *et al.*^89^ Copyright 2018, Wiley-VCH. (E) Reprinted with permission from Wang *et al.*^103^ Copyright 2021, Wiley-VCH. (F) Reprinted with permission from Zhao *et al.*^98^ Copyright 2021, Royal Society of Chemistry.

The earliest use of self-protection methods to achieve the RTP of CDs dates back to 2014, when Yan *et al.*^86^ employed citric acid and ethylenediamine as carbon and dopant sources to achieve the RTP of CDs, but the phosphorescence lifetime at that time was only 160 ms, and thus this result did not attract widespread attention. Until 2016, Chen *et al.*^87^ achieved the RTP of CDs in one step using PVA and ethylenediamine as reaction precursors, with a phosphorescence lifetime of up to 13.4 ms. At this time, the RTP of CDs gradually came into the limelight, and the research frenzy gradually surged. Later, Jiang *et al.*^131^ by simple heat treatment of ethylenediamine and phosphoric acid could produce unexpectedly long room temperature phosphorescence with a duration of about 10 s and a lifetime of 1.39 s (Fig. 4A). It is suggested that the doping of N and P elements is the primary factor in achieving the RTP of CDs. And then, Long *et al.*^91^ reported a new type of fluorine–nitrogen co-doped CDs, which were obtained by a one-step solvent heat treatment, exhibiting excellent water solubility and blue fluorescence in solution or on filter paper, together with pH-responsive green self-protective RTP (Fig. 4B). The spatial protection of C–F bonds and hydrogen bonds was found to reduce the quenching of RTP by oxygen at room temperature, which is key to achieving RTP in CDs. In addition, Wang *et al.*^94^ have successfully engineered a one-step approach for the synthesis of gram-scale CDs with up to 41% total QY by utilizing the polymerization, deamination and dehydration reactions of urea and phosphoric acid in an aqueous environment (Fig. 4C). It was similarly demonstrated that doping with N and P was responsible for achieving white light emission. Also, Jiang *et al.*^89^ reported a facile, rapid, gram-scale preparation of ultra-long RTP CDs by employing microwave-assisted heating of aqueous solutions of ethanolamine and phosphoric acid (Fig. 4D). Further studies showed that the amorphous polymer-like structure of the CDs, intraparticle hydrogen bonding and the presence of doping elements N and P were the major factors responsible for their ultra-long RTP. Furthermore, Wang *et al.*^103^ proposed a thermally driven amorphous-crystalline phase transition-based strategy to achieve multicolor CPDs with emission colors adjustable from green to orange-red. Further studies have shown that self-protected covalent crosslinking framework formation as well as the co-doping of multiple heteroatoms play a crucial role in the generation of RTP CDs. The color tunability of RTPs can be attributed to the different crystalline contents of conjugated π-domains within the CPDs (Fig. 4E). Similarly, Zhao *et al.*^98^ demonstrated that efficient blue-green fluorescent–phosphorescent double-emitting CDs could be easily obtained *via* solvent-free pyrolysis of hydroxyurea (Fig. 4F), and it was demonstrated that the efficient double-emitting properties of the fluorescent–phosphorescent CDs were derived from the aromatic carbonyl group at their edges. In summary, co-doping (*e.g.* N and P co-doping or N and F co-doping) strategies are one of the most effective means applied to achieve ultra-long and efficient synthesis of RTPs with self-protected CDs. In addition, the rational use of edge groups (carbonyl groups) is also expected to achieve self-protected RTP CDs.

## 3. Annual representative studies on CD-based RTP materials

**Fig. 5:**
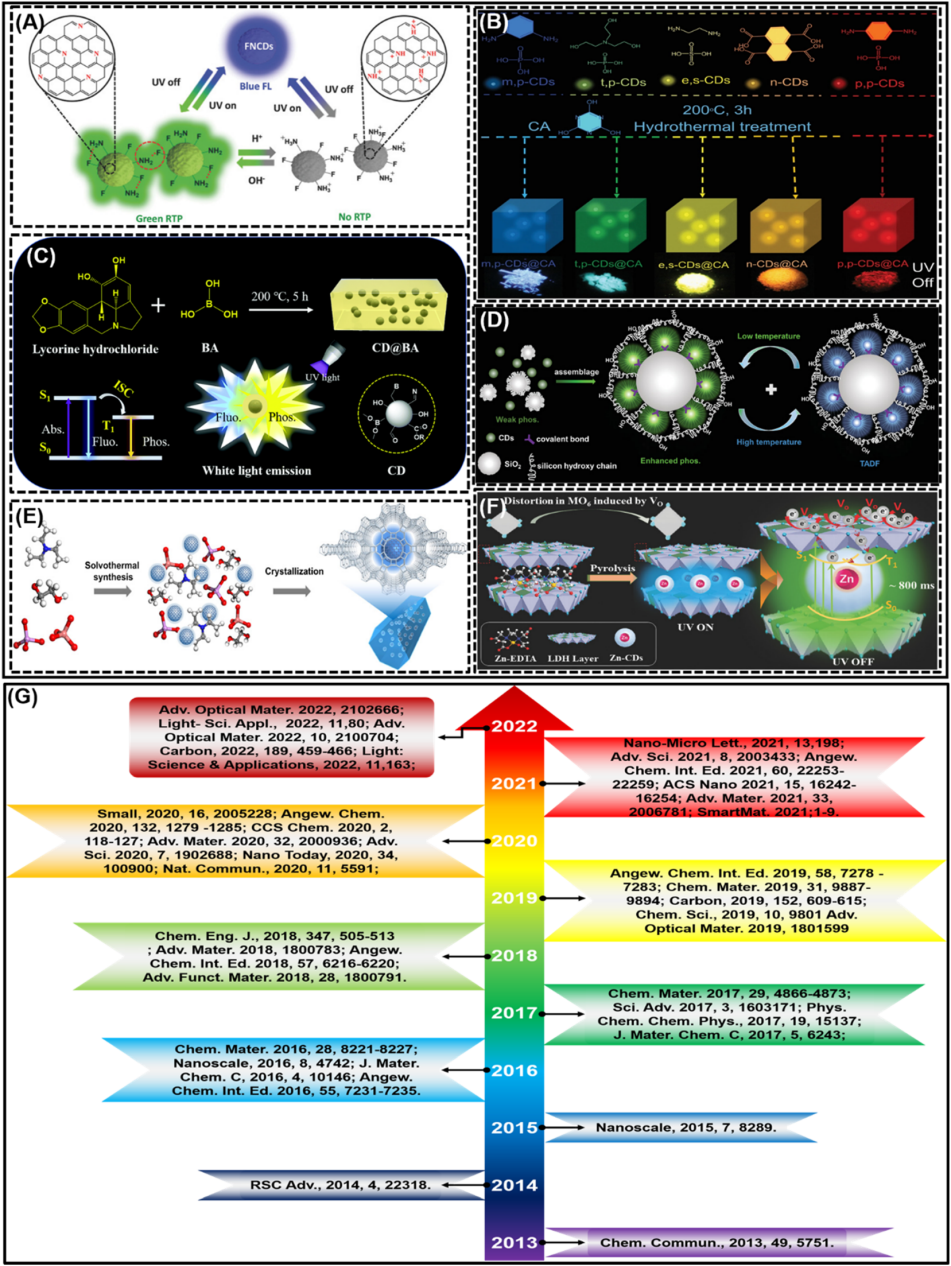
(A) Reprinted with permission from Long *et al.*^91^ Copyright 2018, Wiley-VCH. (B) Reprinted with permission from Zheng *et al.*^74^ Copyright 2022, Wiley-VCH. (C) Reprinted with permission from Li *et al.*^77^ Copyright 2021, Royal Society of Chemistry. (D) Reprinted with permission from He *et al.*^55^ Copyright 2020, Wiley-VCH. (E) Reprinted with permission from Liu *et al.*^63^ Copyright 2017, Science. (F) Reprinted with permission from Shi *et al.*^51^ Copyright 2018, Wiley-VCH. (G) Representative studies of CD-based RTP materials in each year.

According to the synthetic methods described earlier, the synthesis of CDs RTP has been a positive achievement, with progress being made every year. In each year of work, different matrices or methodologies have been employed to fabricate high performance RTP CDs, and the corresponding structures of the resulting RTP CDs varied. Examples include the use of doping to form covalent bonds to achieve RTP of CDs by self-protection^91^ (Fig. 5A), the use of cyanuric acid^74^ (Fig. 5B) and boric acid^77^ (Fig. 5C) as substrate-assisted methods to form rigid structures to encapsulate immobilized CDs to achieve RTP, the use of silica^55^ (Fig. 5D) and zeolites^63^ (Fig. 5E) as mesoporous media materials to encapsulate immobilized CDs to achieve RTP, and the use of layered double hydroxides^51^ (Fig. 5F) as media to immobilize CDs by intercalation to achieve RTP. So far, some significant achievements about RTP CDs have been realized and are shown in Fig. 5G. All these studies were outstanding contributions and prominent representatives in that year, but they were not ranked in the same year. In 2013, Deng *et al.*^42^ employed PVA for the first time to reveal the phenomenon of RTP in CDs, indicating that the hydrogen bonding of the PVA matrix and the C=O bonding on the surface of CDs can achieve cross-linking to form a stable rigid structure, which in turn facilitates the achievement of RTP. In 2014, Yan *et al.*^86^ first observed the RTP of water-soluble CDs without a deoxidizer and other inducers in pure aqueous solution. They found that due to non-radiative electron transfer, phosphorescent emission can be quenched in the presence of iron ions (Fe^3+^), and then turned on by phosphate ions through strong interaction. In the presence of CD-based RTP, it is possible to easily avoid the interference of deoxidizers and other inducers necessary in conventional RTP detection, as well as self-fluorescence and composite matrix scattering light encountered in fluorescence spectrometry. In 2015, Gui *et al.*^133^ for the first time reported RTP logic gates involving RTP emission based on carbon materials. They used capture ssDNA (cDNA) modified CDs and graphene oxide (GO). That is, firstly, the cDNA was combined with carboxyl group surface modified CDs (HOOC-CDs) by carbodiimide chemistry, and then the cDNA-CD conjugates were adsorbed on the surface of GO through π–π stacking interactions to form a cDNA-CD/GO complex. They verified the feasibility of the RTP logic gate based on the cDNA-CD/GO system. The logic gate is simple, easy to operate, and low-cost, does not require complex labeling and modification, and can be efficiently used in actual samples. In 2016, Jiang *et al.*^111^ reported for the first time triple-mode emission (*i.e.*, photoluminescence (PL), up-conversion PL and RTP) on luminescent CDs prepared by compounding *m*-phenylenediamine and PVA. It is worth noting that this work is the first time to realize simultaneously triple-mode emission with a single material and put forward the advanced anti-counterfeiting of a triple authentication concept. And Li *et al.*^47^ synthesized a highly efficient CD-based phosphorescent material by heating a mixture of urea and nitrogen doped CDs (NCDs) using one pot. It was found for the first time that new energy structures can be generated by C=N bonds on the NCD surface, and evidence that they are the origin of phosphorescence is proposed. Then, Tan *et al.*^49^ prepared simple and large-scale nitrogen doped carbon quantum dots (N-CQDs) under microwave irradiation which was a new strategy to synthesize N-CQDs *via* using isophorone diisocyanate (IPDI) as a single carbon source. N-CQDs were dispersed in the polyurethane (PU) matrix, under the excitation of ultraviolet (UV) light, and they emitted not only FL, but also phosphorescence and DF at room temperature. In the following year, amphiphilic carbon quantum dots (ACDs) were also successfully prepared by Tan *et al. via* treating oil-soluble N-doped carbon quantum dots by a one pot hydrothermal method.^134^ The obtained ACDs may be homogeneously dispersed in PVA and the PU matrix. Furthermore, RTP can be recognized in these ACD based composites. This is the first time that ACDs realize phosphorescent emission in polymers, which greatly broadens the research and application scope of CQDs. In 2017, Chen *et al.*^87^ reported for the first time the aggregation-induced RTP of self-quenching-resistant nitrogen doped CD powder by structure design *via* using PVA-chains, and the potential application of the temperature sensor is preliminarily prospected. Jiang *et al.*^53^ also reported a new method for preparing room temperature long afterglow materials by covalently fixing CDs onto colloidal nanosilica (nSiO_2_). A CD-based long afterglow material (*i.e.*, m-CDs@nSiO_2_) is reported for the first time, and the material is suitable for room temperature, and can even be directly observed in an air-saturated aqueous medium. The results show that the long afterglow materials of m-CDs@nSiO_2_ have mainly delayed fluorescence properties and mixed partial phosphorescence. In addition, Joseph *et al.*^62^ also showed the observation of RTP emitted by CDs embedded in a silica gel matrix at room temperature. CDs in silica gel showed a long phosphorescence lifetime of 1.8 s, which is the highest value of CDs in solid state matrices, and the phosphorescent emission is displayed in the white gamut region in the chromaticity diagram. As a type of inorganic porous material, zeolite has emerged as one of the most desirable materials for loading and encapsulating metal nanoparticles and luminescent quantum dots because of its three-dimensional ordered structure and strong thermal stability. In the same year, Liu *et al.*^63^ embedded CDs in a family of zeolitic crystalline matrices *in situ* under solvothermal/hydrothermal conditions to prepare a novel category of CD-based TADF materials with ultra-long lifetimes and achieved high quantum yields (QYs) up to 52.14%. This work offers a new “dots-in-zeolites” scheme for the design and synthesis of innovative TADF materials, which may open the application of various delayed fluorescence in various fields. In 2018, He *et al.*^43^ used electrospinning technology to incorporate CDs into PVA, and achieved the two goals of RTP and TADF. It was found for the first example of the CDs/polymer system that the ordered mesoporous structure of electrospun CDs/PVA nanofibers allows effective stabilization of the triplet state of CDs, thus realizing the RISC process of TADF. To date, this is the first time that TADF has been found in the CDs/polymer system. Jiang *et al.*^131^ reported the first example of conversion of a fluorescence material to RTP with an external heat stimulus, that is using ethylenediamine and phosphoric acid as raw materials, fluorescent emissive polymer CDs were prepared by simple heat treatment and can produce unexpected ultra-long RTP. It was found that the doping of N and P elements may be critical to the RTP production of CDs. Similarly, Jiang *et al.*^89^ again reported an ultra-long CD-based RTP material by microwave-assisted heating of an aqueous solution of ethanolamine and phosphoric acid which showed the longest RTP lifetime (1.46 s) for CD-based materials to date. Long *et al.*^91^ reported for the first time fluorine–nitrogen co-doped CDs (FNCDs) possessing long-lived triple excited states which emit pH-stabilized blue fluorescence and pH-responsive green self-protected RTP. In 2019, Li *et al.*^54^ proposed a reasonable strategy for a kind of silica-based composite using CDs (CDs@SiO_2_) in air saturated aqueous media for realizing ultra-long RTP. More importantly, they reported the first application in practical imaging of biological samples for CD-based RTP materials. Also, through one-step heat treatment of nitrogen doped CDs and boric acid (BA) (N-CDs/BA), a universal method for activating RTP of both heteroatom-free and heteroatom-containing CDs was achieved.^120^ N-CDs/BA exhibits the best phosphorescence lifetime of 2.26 s and a PQY of 17.5%, representing the most advanced record for CD-based RTP materials to date. In addition, Su *et al.*^92^ proposed a method of microwave synthesis of nitrogen and phosphorus co-doped CDs (P-CDs) *via* using triethanolamine as the carbon source and phosphoric acid as the dopant. The prepared P-CDs showed not only bright-blue fluorescence in aqueous solution, but also obvious green phosphorescence on filter paper. More importantly, this study successfully realized a dual-channel signal for the detection and analysis of the pH value by using P-CDs for the first time, and found that the RTP signal is more sensitive than the fluorescence signal, and thus may provide a wider linear range. In the same year, Yuan *et al.*^135^ also demonstrated for the first time that the synthesized single-component white carbon nitride quantum dots (W-CNQDs) exhibit a double emission of blue-yellow fluorescence–phosphorescence. The W-CNQDs offered an overall photoluminescence quantum efficiency (PLQY) of 25%, which is the largest of any white light-emitting material reported so far. In 2020, using CD modified amorphous silica (CDs-SiO_2_) as the raw material, He *et al.*^55^ realized RTP and TADF in the solid state and aqueous solution through a one-pot sol–gel methodology without the addition of any heavy atomic scramblers and the removal of dissolved oxygen. Importantly, the thermoluminescence spectra of the CD-based matrix were for the first time visualized and evaluated. Jiang *et al.*^136^ reported for the first time a simple method to prepare CDs (*i.e.*, TA-CDs) with double emission, robustness and aggregation induced RTP properties. The study showed that the yellow RTP of TA-CD powder may be due to its aggregation. Liang *et al.*^58^ used silica to confine water-soluble phosphorescent carbon nanodots (WSP-CNDs@silica) in their nanospace and realized the ultra-long and efficient phosphorescence of CNDs. The phosphorescence lifetime and quantum yield (QY) reached 1.86 s and 11.6%, respectively, which is the best value of water-soluble phosphorescence nanoparticles reported so far. It is shown that the silica shell outside of CDs restricts the rotation and vibration of the bonds in CDs, resulting in the long life and high efficiency phosphorescence of CDs. Park *et al.*^76^ reported a new engineering approach to manage singlet-triplet energy splitting (Δ*E*_ST_) in graphene quantum dots (GQDs)/graphene oxide quantum dots (GOQDs) through varying the ratio of oxygen-containing carbon to sp^2^ carbon (*γ*_OC_). This is the first time that GQDs are used as a long-life RTP and TADF material to demonstrate anti-counterfeiting and multi-level information security. Wang *et al.*^94^ developed a one-step synthesis method with low cost, rapid processing and environmental protection for the fabrication of single-component white luminescence carbonated polymer dots (SW-CPDs) on a gram scale with high efficiency. The overall QY was as high as 41%, and that was the largest value ever recorded for solid-state fluorescent CDs. The results showed that the hybrid fluorescent/phosphorescent components promoted the emergence of white light emission. In addition, Zhang *et al.*^66^ successfully achieved high-efficiency afterglow CDs@zeolite composite materials by simply grinding the solid raw material zeolite and precursor CDs at room temperature and then performing thermal crystallization of CDs@zeolite by a solvent-free thermal synthesis strategy. In this method, CDs are embedded into the growing zeolite crystals with the maximum extent, and the non-radiative transition of CDs is surpassed by the strong host–guest interaction, and thus composite materials with ultra-long double emission of TADF and RTP are prepared, and have a lifetime of 1.7 s and 2.1 s, respectively, as well as a QY of 90.7% and a PQY of 24.4%, respectively. These values are at the top superiority level among CD-based PL materials, and thus, represent the majority of organic afterglow materials. What's more, in 2021, Li *et al.*^79^ reported a method to prepare highly efficient RTP materials from crystalline heat-annealed CDs and BA composites which can induce amorphous to crystalline transition by grinding. This method can enable CDs to be uniformly embedded in BA crystals to the maximum extent, reduce the non-radiation attenuation of CDs, and promote the cross-connections between systems by suppressing the free vibration of CDs, thus generating strong RTP materials. The reported PQY is the highest (48%). It is well known that water-soluble red afterglow imaging agents have a great penetration depth and nondurable excitation characteristics which have potential application prospects in time-gated afterglow bioimaging. Thus, Liang *et al.*^59^ reported a red afterglow imaging agent composed of Rhodamine B (RhB) and CNDs, which were confined in a hydrophilic silica shell to form CNDs-RhB@silica nanocomposites. CNDs-RhB@silica can achieve a luminescence lifetime and afterglow QY of 0.91 seconds and 3.56%, respectively, which is the best result for the red afterglow region. Liang *et al.*^60^ also reported a time division duplex technology based on environmentally friendly CNDs with controllable luminescence lifetime. It was the first time they demonstrated that the time-division duplexing technique for CNDs and CNDs@silica is independent of the emission color and intensity depending on the phosphorescence lifetime under control. Water has been demonstrated to play an essential role in modulating the luminescence lifetime of CNDs by quenching triple excitons. In addition, Tan *et al.*^104^ also reported a method to realize time-dependent phosphorescence colors in CDs, which were synthesized through a one pot hydrothermal method by using levofloxacin as the raw material. They realized a new type of time-dependent phosphorescence color that changed from orange to green in a very short time (1 s). In 2021, Zheng *et al.*^137^ also reported a general approach in which effective radiative energy transfer can be applied to support the reabsorption of upconversion materials (UMs) into CD-based room temperature near-infrared excited multicolor afterglow materials (CDAMs). Please note that this is the first report on the multicolor afterglow of near-infrared excited in CDAMs. More importantly, this work provides a general route for constructing novel room-temperature afterglow materials with tunable excitation wavelengths. For the first time, Zhou *et al.*^138^ proposed an approach for efficient energy transfer mediation to boost the RTP of CDs by incorporating pure phosphorescent CDs into the afterglow matrix. In this system of design, there is a significant increase in the emission intensity, RTP lifetime and emission time of CDs, and all CD-based materials emit visible phosphorescence for more than 20 seconds after UV excitation. In addition, Mo *et al.*^139^ provided the first example of visible light excited TADF in aqueous solution by confining the co-doping of CDs with fluorine and nitrogen in silica nanoparticles (F, NCDs@SiO_2_). Although there have been many reports on RTP materials, it is still a challenging task to realize ultra-long RTP in aqueous media, especially for CD-based materials. In 2022, Jiang *et al.*^73^ developed a robust organic long persistent luminescence (OLPL) system with hour-level afterglow emissions through simple microwave-assisted heating of a mixture of m-CDs and urea. It is a very uncommon case of an OLPL system displaying hourly afterglow under ambient conditions, even for aqueous media. Further studies showed that the generation of covalent bonds between cyanuric acid and CDs played a key role in the afterglow presentation. Green preparation has always been the synthetic route pursued by everyone. At present, RTP materials are greatly developed. Nevertheless, it is a huge priority to achieve both multicolor and long wavelength RTP emission with favorable stability in CD-based RTP materials. Liang *et al.*^140^ proposed a new and general “CDs-in-YOHF” scheme to yield multicolor and long wavelength RTP by confining different CDs to a Y(OH)_*x*_F_3−*x*_(YOHF) matrix. It should be noted that the RTP lifetime of the orange emissive CDs-o@YOHF is the longest in the reported single-CD-matrix composite materials with emission above 570 nm. Compared with the common representative matrices, the YOHF matrix is also proved to be more effective in protecting the long-wavelength triplet emission of CDs-o. Similarly, Mo *et al.*^61^ reported a newly developed strategy to incorporate phosphorescent CD and fluorescent dyes into monodisperse silica nanoparticles below 20 nm to achieve multi-color long afterglow in aqueous solution. For the first time, CD-based multicolor long afterglow systems (green, yellow, orange and red) were fabricated in aqueous solution by cascaded Förster resonance energy transfer. Especially, under UV excitation, a prolonged red afterglow with a Stokes shift of 255 nm was developed. So far, there have been few studies on thermal stimulated response photoluminescence of CD-based materials. Xu *et al.*^141^ reported for the first time a polymeric nanocomposite incorporating fluorinated CDs (FCDs) that can be efficiently synthesized in large quantities through the utilization of commercial water-soluble polymer sodium carboxymethylcellulose (CMCNa) as a stable matrix. The synthesized FCDs–CMCNa has bimodal emission, *i.e.*, both solid state fluorescence and in-room RTP. It is more interesting to note that FCDs–CMCNa exhibits special temperature-sensitive optical properties when the temperature is reduced from 300 K to 90 K. It exhibits an increase in fluorescence/phosphorescence intensity with decreasing temperature up to the switching point of 150 K, which then gradually decreases with decreasing temperature to 90 K. Later, Liu *et al.*^142^ proposed a series of flexible dynamic ultra-long RTP polymer composites, which are illuminated by halogen doped CDs and have fully programmable emission. They had produced a transparent, flexible and fully programmable dynamic ultra-long RTP composite film with reliable gray-scale display ability from the synthesized RTP material for the first time. As can be seen, each year's contribution has grown year on year, indicating a steady stream of scholars working on CD-based RTP materials with notable achievements. In each year of the prominent work represented, RTP of CDs was slowly developed in the initial stage, however, three years later, it was springing up. This indicates that as research progresses, a steady stream of findings is discovered that continue to advance the development of RTP for CDs.

**Fig. 6:**
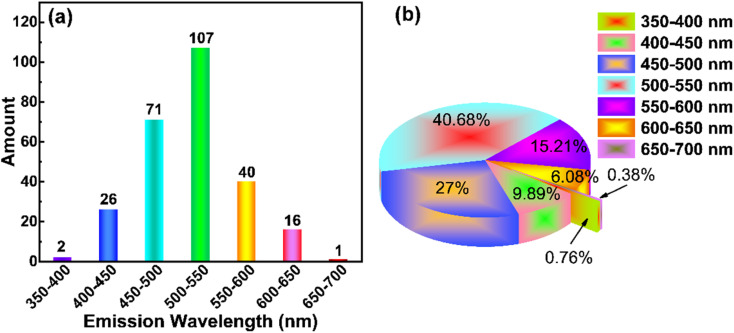
(a) Distribution of phosphorescence emission of CD-based RTP. (b) The proportion of phosphorescence emission of CD-based RTP in the total number of reported articles.

To sum up, the outstanding work of CD-based RTP materials is growing every year. According to Fig. 2, we conducted statistical analysis on all the 166 retrieved papers and summarized the corresponding RTP emission wavelengths (it is worth noting that the number of wavelengths counted here is more than the number of papers published because some articles reported multicolor wavelengths), as shown in Fig. 4. According to the statistical results, the emission of CD-based RTP materials is mainly concentrated in the range of 500–550 nm (Fig. 6a), accounting for 40.68% of the total (Fig. 6b). In other words, the current CD-based RTP materials mainly emit green light. The second is blue to green emission with emission wavelengths concentrated between 450 and 500 nm, followed by yellow-orange emission with emission wavelengths concentrated between 550 and 600 nm. It can be seen that the achievable emission of CD-based RTP materials is mainly concentrated in the short wavelength (450–600 nm) region of visible light, while reports of long wavelength emission (>600 nm) are very rare. Certainly, the realization of long-wavelength emission from CD-based RTP materials, even extending into the near-infrared region, is the focus of current research and the key to enhancing and broadening their application areas. This is a challenging research problem which needs to be solved.

## 4. Annual representative studies of phosphorescence lifetime in CD-based RTP materials

**Fig. 7:**
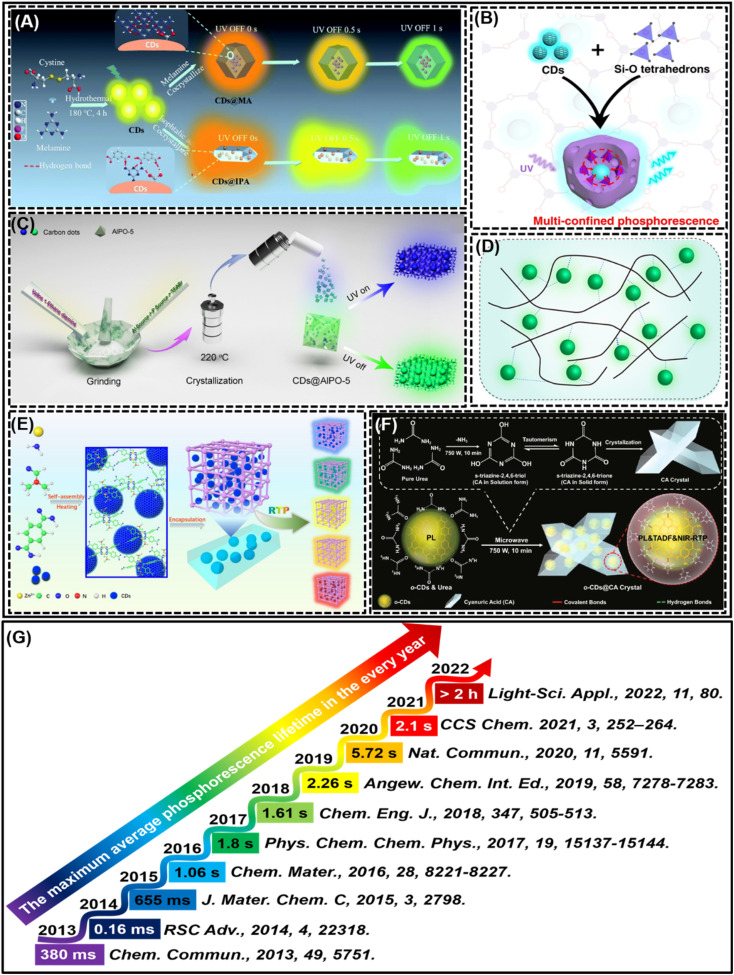
(A) Reprinted with permission from Qu *et al.*^125^ Copyright 2021, Royal Society of Chemistry. (B) Reprinted with permission from Sun *et al.*^57^ Copyright 2019, Springer. (C) Reprinted with permission from Zhang *et al.*^66^ Copyright 2020, Chinese Chemical Society. (D) Reprinted with permission from Cao *et al.*^45^ Copyright 2022, American Chemical Society. (E) Reprinted with permission from Xu *et al.*^80^ Copyright 2022, American Chemical Society. (F) Reprinted with permission from Wang *et al.*^72^ Copyright 2021, Springer. (G) The longest phosphorescence lifetime of CD-based RTP materials in each year.

RTP materials have attracted much attention because of their great optical application potential. With the deepening of research, researchers have made breakthroughs in CD-based RTP materials, such as multi-mode emission RTP materials,^72,111^ multi-color RTP materials,^48,75,120,140,143^ aqueous solution RTP materials,^55,59,61,86^ multifunctional detection application RTP materials,^43,92,134^ biocompatible RTP materials^54^ and high PQY^79,94,135^ and ultra-long phosphorescence life RTP materials.^46,57,58,73,89,131^ However, it is still difficult to obtain RTP materials with simultaneous long-lifetime and high PQY. In addition, phosphorescence lifetime is one of the important parameters to measure CD-based RTP materials. Phosphorescence lifetime is a key feature of RTP materials, and ultra-long phosphorescence lifetime is central to the excellent performance of RTP materials. The key to achieving long phosphorescence lifetimes is the choice of the RTP material structure or the implementation of methods, such as the use of melt co-crystallisation^125^ to immobilize CDs in the form of wrapping to achieve RTP (Fig. 7A), the utilization of rigid structural networks and the coexistence of covalent bonds^57^ to immobilize CDs in a three-dimensional spatially confined way to achieve RTP (Fig. 7B), the adoption of porous space crystalline materials^66^ to immobilize CDs by adsorption and intercalation to achieve RTP (Fig. 7C), the employment of polymers to covalently cross-link CDs^45^ to achieve RTP (Fig. 7D), the employment of metal–organic frameworks with porous structures providing active sites^80^ to immobilize CDs to achieve RTP (Fig. 7E), and the utilization of high strength rigid crystal structures^72^ to immobilize CDs to achieve RTP (Fig. 7F). Each year's work has made corresponding outstanding contributions, from the initial millisecond level to the later second level, and then to the current hour level, which are major breakthroughs one after another. As shown in Fig. 7G, the best phosphorescent life of CD-based phosphorescent materials is shown in each year from the first discovery to the present. In 2013, Deng *et al.*^42^ reported for the first time that the phosphorescence phenomenon of CDs in the PVA matrix was observed, and its average phosphorescence life was as high as ∼380 ms. Then, in 2014, Yan *et al.*^86^ reported for the first time that the phosphorescence of CDs in pure aqueous solution was observed, but its average phosphorescence lifetime was only 0.16 ms. In 2015, Deng *et al.*^46^ embedded CDs into a KAl (SO_4_)_2_·*x*(H_2_O) matrix to prepare composite powder of CDs, and presented long-lived RTP with an average phosphorescence life of up to 655 ms. In 2016, Li *et al.*^47^ prepared an efficient CD-based phosphorescent material, that is, NCDs were heated in one pot by treating a mixture of urea and NCDs, and then they were incorporated into the composite matrix. The resulting material had an ultra-long phosphorescent life of 1.06 s under the excitation of 280 nm. In 2017, Joseph *et al.*^62^ embedded CDs in a silica gel matrix to obtain an RTP material. The CDs showed strong blue fluorescence in aqueous dispersion and showed green afterglow when combined with silica gel. Under the excitation of 380 nm, the phosphorescent emission lifetime of the CDs is about 1.8 s, which is the highest value of CDs in a solid matrix. In 2018, He *et al.*^43^ combined CDs with PVA *via* the help of electrospinning technology, and realized RTP and TADF at the same time. In addition, these nanofibers showed a longer average afterglow lifetime of 1.61 s and a visual recognition period of 9 s. In 2019, Li *et al.*^43^ realized the preparation of RTP of CDs with or without heteroatom doping through a single-step thermal treatment of CDs and boric acid. This composite exhibits the highest phosphorescence lifetime of 2.26 s and a PQY of 17.5%, and this is the maximum record for CD-based RTP materials to date. In 2020, Sun *et al.*^57^ obtained RTP materials with ultra-long life, high-level PQY and superior stability by rational design and fabrication of multi-constrained CDs. The designed multi-constrained CDs possess an ultra-long lifetime of 5.72 seconds, 26.36% PQY, and outstanding stability to strong oxidants, acids, bases, and polar solvents. In 2021, Yu *et al.*^68^ systematically adapted the reaction parameters and host–guest interactions to achieve nine green RTP CDs-in-zeolite (CDs@zeolite) composites with engineering lifetimes ranging from 0.38 to 2.1 s under solvent-free conditions. In 2022, Jiang *et al.*^73^ developed the first CD-based organic long persistent luminescence system with a duration of more than 1 h. A system based on CDs (named m-CDs@CA) is reported, which can be conveniently and efficiently fabricated through the utilization of a household microwave oven. What is even more impressive is that its long-term sustained luminescence may be noticed under ambient conditions, even in aqueous media. In summary, the extension of phosphorescence lifetimes has always been at the heart of scholars' pursuits, and shows that phosphorescence lifetimes occupy an irreplaceable and central position in the RTP of CDs, and are a sign that CDs exhibit RTP properties. Progress in the field of research can be seen in the representative work each year, and in the advancement of scientific capabilities. Following on from the previous research, the phosphorescence lifetime of CD RTP has made a qualitative breakthrough, extending from the initial millisecond level to the hourly level, which provides favorable preconditions for the practical application of CDs RTP.

**Fig. 8:**
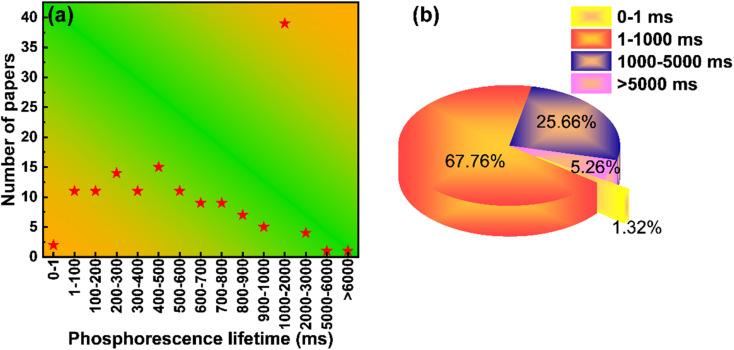
(a) Distribution of phosphorescence lifetime of CD-based RTP. (b) The proportion of phosphorescence lifetime of CD based-RTP in the total number of reported articles.

Phosphorescence lifetime is an important index to measure RTP materials, and the phosphorescence lifetime of CD-based RTP materials is also constantly developing. According to Fig. 2, the phosphorescence lifetime of the published CD-based RTP materials was statistically analyzed. As shown in Fig. 8a, CD-based RTP materials with different phosphorescence lifetimes have been reported one after another, but the main phosphorescence lifetimes are between 1 and 1000 ms. More obviously, the phosphorescence lifetime of most CD-based RTP materials is still at the millisecond level. Of course, there are also some outstanding performances, such as the existence of phosphorescence lifetime at the level of hours, but this is rare. According to the analysis of statistical data, the ratio of phosphorescence lifetime between 1 and 1000 ms accounts for 67.76%, which is a very high percentage, while the ratio between 1000 and 5000 ms reaches 25.66% (Fig. 8b). It can be seen that the preparation of CD-based phosphor materials with second phosphorescence lifetime is still in the stage of development. It is worth noting that CD-based RTP materials with all second phosphorescence lifetimes account for less than half of all reported CD-based RTP materials. This is a very terrible existence. Therefore, developing CD-based RTP materials with ultra-long phosphorescence lifetime is a hot spot and a difficult problem at present.

## 5. Applications

Realizing the final application is the value of a material. As new luminescent materials, CD-based RTP materials are no exception. First of all, some CD-based materials have the characteristics of DF, which have the property of long life and can be used in the field of information security without prompt excitation. Secondly, some CD-based materials with DF characteristics have also been accompanied by RTP performances, and such multiple afterglow emission offers additional possibilities for information encryption and anti-counterfeiting applications. In addition, RTP materials based on CDs have strong sensitivity characteristics in some special detection, and are also used for response detection of ions, pH, temperature, time, fingerprint and so on. It is worth noting that CD-based RTP materials also have low toxicity and good biocompatibility, and also have great potential and development in the field of biological imaging. More importantly, CD-based RTP materials fully embody the characteristics of light-emitting materials, have good tunability, luminescence, stability and other optical properties, and have been greatly developed in the field of light-emitting diodes (LEDs). Table 3 summarizes some main applications of CD-based RTP materials. In addition, the special properties of CD based-RTP materials, such as excitation-related, temperature-related and afterglow color adjustable properties, enable them to build multi-level security defense in the field of information encryption and anti-counterfeiting. The long afterglow life of CD based-RTP provides new possibilities for easier identification of authenticity and information content. It allows significantly increased time to visualize cell states, enables speedy detection, and also facilitates bioimaging and detection. Generally speaking, the RTP material based on CDs possesses huge application prospects in the field of anti-counterfeiting,^110,126,127,144–150^ information encryption,^113,115,118,119,121,123,128,151–160^ sensor detection,^114,124,129,130,147,161–174^ LEDs,^175–179^ biological imaging,^134,180,181^ logic gates,^133^ fluorescent ink^112,182–184^ and other fields^116,117,122,185–195^ with the properties of DF, TADF, long afterglow life and adjustable afterglow color.

**Table 3:** Applications of CD-based RTP materials

Application		Method	Afterglow emission (nm)	Afterglow lifetime (*τ*) ms^−1^ (298 K)	Remarks	Ref.
Anti-counterfeiting		A composite film of m-CDs (CDs prepared from *m*-phenylenediamine) and polyvinyl alcohol (PVA)	500	456	CDs–PVA	111
		Microwave-assisted heating of ethanolamine and phosphoric acid aqueous solution	535	1460	URTP CDs	89
		*In situ* in the hydrothermal system by using an organic amine as the template and the precursor of the CDs and an inorganic crystalline framework as the matrix	620	10.94	CDs@MnAPO-CJ50	81
		By one-step heat treatment of CDs and boric acid (BA)	490–570	2260	a-CDs/BA	120
		One-step solvothermal treatment	455	1210	FNCDs	91
		Hydrothermal treatment of trimellitic acid	560	183.6	TA-CDs	136
		A hydrothermal reaction of succinic acid and diethylenetriamine	500–575	880	MP-CDs	144
		Thermal-treated CDs into a PVA matrix	420–440	>100	CD@PVA	113
Information encryption		A hydrothermal treatment of nSiO_2_ solution mixed with m-CD solution	470	703	m-CDs@nSiO_2_	53
		Using polyacid and diamine to synthesize PCDs through a one-step hydrothermal treatment	494	658.11	PCDs	88
		A further heat treatment of the F-CD powder at a higher temperature	538	1390	F-CDs	131
		By *in situ* embedding CDs within a series of zeolitic crystalline matrices under solvothermal/hydrothermal conditions	430	350	CDs@zeolite	63
		By utilizing CDs and melamine to construct hydrogen-bonded networks to form a polymer	529	664	M-CDs	82
		By pyrolysis of citric acid and boric acid precursors	466–638	113.9–581.76	B-CD	109
		One-pot hydrothermal treatment of levofloxacin	555/630	354/237	CD@paper	104
Biological imaging		By confining CNDs in a silica encapsulation layer	520	1860	WSP-CNDs @ silica	58
		Neutral red, KNO_3_, MgCl_2_ and KH_2_PO_4_ as precursors *via* the molten salt method	518/520	546/616	CD71	180
		By confining the fluorine and nitrogen co-doped CDs in silica nanoparticles	500	480	F, NCDs@SiO_2_	139
		One-step solvent-free catalytic assisted strategy	554	243.2	M-CDs	108
Light-emitting diodes	WLED	A one-pot heat treatment of a mixture of urea and NCD	490	1060	HN powders	47
	LEDs	CD-based composites were prepared by simply mixing CD solution and NaOH under constant stirring	480	705	CD/CA	70
	LEDs	*In situ* solvent-free thermal crystallization	530	2100	CDs@AlPO-5	66
	WLED	One-step heat treatment of lycorine hydrochloride and boric acid	520	1760	CD@BA	77
	WLED	A one-step heat treatment process of urea and phosphoric acid aqueous solutions	495	320	SW-CPDs	94
	WLED	Encapsulating multicolor lignin-derived CDs and PVA into a delignified wood framework	505	208.83	LTW	176
	WLED	Through the solvothermal reaction of polyvinylpyrrolidone (PVP), urea, and seed CDs	501–596	419	CD powder	143
Sensing detection	pH and Fe^3+^	One step hydrothermal	440	0.16	CD-based RTP	86
	pH	Microwave strategy to synthesize P-CDs by using triethanolamine serving as the carbon source with phosphoric acid	518	820	P-CDs	92
	Fe^3+^	*In situ* green synthesis of CDs by using Schisandra chinensis polysaccharide as the only carbon source	510	271.2	CD/PVA	114
	Humidity	By combining chelate and hydrothermal methods	525	1.34	Eu–CDs/PV	186
	Humidity	By embedding biomass derived CDs into cellulose fibrils	560	167.31	CDs@Cellulose	85
	Fe^3+^	The CD solution mixed with CA under constant stirring	480	687	CD–CA	71
	O_2_	By intercalating the CD precursor (EDTA) into the LDH interlayer	525	386.8	CDs-LDHs	50
Temperature sensing		Hydrothermally synthesized using PVA and ethylene diamine	564	13.4	NCD	87
		By direct calcination of carbon sources (1,2,4-triaminobenzene) with inorganic salts	506	1280	CDs@MP	124
		By incorporating CDs into poly(vinyl alcohol) (PVA) with the assistance of electrospinning technology	569	1610	CDs/PVA	43

However, the application of CD RTP is also limited by its own deficiencies. First, the clarity in the luminescence mechanism of CDs is lacking. Different types of CDs have been reported one after another since the development of CDs, but equally inconsistent types of luminescence mechanisms have emerged, which seriously affects the unified definition and future application of CDs and prevents a systematic unification. Secondly, the process for the preparation of RTPs for CDs is too harsh. The current implementation of RTP for CDs requires the assistance of a matrix, high temperature or multi-step synthesis, and the process requirements are too demanding and costly, limiting their preparation and application for large scale production. Then, the RTP emission wavelengths of CDs are too short-term. According to the reported articles, most of the RTP emission wavelengths of CDs are concentrated in the range of 480–550 nm, while there is relatively little RTP emission for long wavelengths (>550 nm), which severely limits the application of RTP characteristics of CDs for advanced information security and encryption. Finally, the PLQYs for CDs RTP are relatively low. There is an urgent need for green preparation of RTP CDs with high quality and high PLQY, especially with long wavelength emission. In the field of optoelectronic devices, especially for WLED applications, red emission is the key to achieving efficient WLEDs and strong luminescence is also the key factor to enhance device performance, and the emission of current RTP CDs greatly hinders their application in optoelectronics. Thus, the current low PLQY and the emission of RTP CDs have greatly hindered their application in the field of optoelectronics.

## 6. Challenges and prospects

Although significant achievements in the synthesis and diversified application investigations of CD-based RTP composites have been made, there remain enormous challenges to face. The specific aspects are as follows:

First, since the discovery and development of CDs, the luminescence mechanism of CDs has always been questioned since it does not have a unified structure. At present, we can accept two main categories, namely, carbon core luminescence and surface state luminescence. Similarly, the luminescence of CD-based RTP materials, as a special property of CD luminescence, has not yet been clearly explained and defined. For the luminescence mechanism of CD-based RTP, there are different opinions, each with its own words. For example, it has been reported that CD-based RTP originates from C=N, C=O, C=F, hydrogen bonds, polymer matrices and so on. However, these remarks are all unilateral. Thus, it is essential to address systematically their mutual and distinctive features, as well as their structural evolution patterns during CD synthesis. At the same time, the formation process and luminescence mechanism of CD-based RTP are explored and summarized by combining some more advanced characterization technologies and theoretical calculations, so that future generations can better understand the luminescence mechanism of CD-based RTP materials.

Second, luminescence quantum efficiency is one of the important indicators to measure luminescent materials. As a new emerging carbon based fluorescent nanomaterial, the PLQY of CDs has always been a hot and difficult research topic, and improving the PLQYs of CDs has always been our goal. More importantly, the PQY of CD-based RTP materials is much lower than the PLQY of CD fluorescence, which makes improving the PQY of CD-based RTP materials an urgent need. In addition, compared with CD-based RTP materials, the DF characteristics of CDs require a smaller energy difference (between the excited singlet state and the excited triplet state), which will make the preparation of CD-based DF materials more difficult. Because this also requires that the spatial overlap between the HOMO and LOMO be very small, which in turn reduces the radiation transition rate and ultimately leads to a low PQY. However, reasonable structural design, purification and appropriate matrix selection are expected to solve the pressing problem of low PQY of CD-based RTP materials.

Third, the recognition ability of human eyes is limited. In the visible light region, the luminescence of CDs is mainly concentrated in the short wavelength region (<600 nm), and even the luminescence of CD-based RTP materials is mainly concentrated in the blue to green light range. These short wavelength lights are harmful to the human body. In addition, the luminescence of CD-based RTP materials is mainly concentrated in the solid state, and the luminescence in solution is relatively less. These limitations have seriously hindered the application of CD-based RTP materials in multi-color displays and bio-imaging. Therefore, the development of CD-based RTP materials with a long wavelength and liquid phase luminescence has also become a hot spot and an unsolved problem in current research. But, it is expected to solve the problem of luminescence at long wavelengths and in the liquid phase by choosing suitable hydrophilic matrices and surface modification.

Fourth, the phosphorescence lifetime is also one of the important indicators to measure the performance of CD-based RTP materials. Currently, the phosphorescence lifetime of CD-based RTP materials is mainly concentrated at the second level, and it is only a few seconds to several tens of seconds. Regarding the long-lifetime research, there are still few reports on minute-level, and even hour-level phosphorescence lifetime researches. In addition, CD-based RTP materials with an ultra-long phosphorescence lifetime are expected to show application value in new fields, such as solar cells. In order to realize CD-based RTP materials with ultra-long phosphorescence lifetime, an important matrix selection is the key to solving the problem.

Finally, advanced synthesis methods are one of the key technologies for preparing high-quality CD-based RTP materials. At present, the synthesis route of CD-based RTP materials mainly relies on a two-step method, that is, a matrix-assisted synthesis route is adopted, CDs are synthesized first, and then encapsulated into a matrix to realize RTP. This synthesis method is complicated and inefficient. In addition, the stability of the synthesized CD-based RTP material is also not good. More importantly, in addition to cumbersome synthesis methods, complicated purification processes are also required in the process of realizing CD-based RTP, and the low repetition rate of these synthesis routes greatly limits its large-scale commercial application. Therefore, in order to solve a series of problems in the preparation process, one kind of excellent matrix with a simple and fast preparation method and high stability properties is the only choice.

In summary, CD-based RTP materials have many excellent properties such as low cost, low toxicity and tunable luminescence. It has some advantages unmatched by other RTP materials, and has become a rising star in the fields of anti-counterfeiting, information security, LEDs, sensor detection and so on. However, there are still some problems, such as unclear luminescence mechanism, low PQY, short luminescence wavelength, insufficient phosphorescence lifetime, and simple and convenient preparation methods. Therefore, as a new CD-based RTP material in the future, it will have advantages such as economy, liquid phase stability, long wavelength emission, multi-color emission, high PQY and environmental friendliness. At the same time, CD-based RTP materials can also be combined with other high-performance materials to obtain new composite materials and realize application value in new fields. It is hoped that through the continuous attempts and efforts of the broad masses of people, in modern society where opportunities and challenges coexist, CD-based RTP materials are expected to popularize people's lives in the future and become the next generation of newly applied luminescent materials.

## Author contributions

All authors contributed to writing and revision of the manuscript.

## Conflicts of interest

The authors declare no competing interests.

## Supplementary Material
